# Supplementary material for: Synthesis of Novel N-Heterocyclic Compounds Containing 1,2,3-Triazole Ring System via Domino, “Click” and RDA Reactions
Source: Molecules. 2019 Feb 21;24(4):772. doi: 10.3390/molecules24040772 (PMC6412576; doi:10.3390/molecules24040772)

Supplementary Information

Synthesis of novel N-heterocyclic compounds containing 1,2,3-triazole ring system via domino-, "click" and RDA reactions

Márta Palkó[a], Mohamed El Haimer[a], Zsanett Kormányos[a] and Ferenc Fülöp*[a,b]

Address:

[a] Institute of Pharmaceutical Chemistry, University of Szeged, Interdisciplinary excellence centre, Eötvös u. 6, H-6720 Szeged, Hungary.E-mail: fulop@pharm.u-szeged.hu.

[b] MTA-SZTE Stereochemistry Research Group, Hungarian Academy of Sciences, Eötvös u. 6, H-6720 Szeged, Hungary

Contents

1. Copies of ^{1}H- and ^{13}CNMR spectra of **4**-**16** S2
2. HPLC chromatograms of enantiopure domino products (+)-**6**, (-)-**6**, (+)-**15** and (-)-**15** *S10*
3. HPLC chromatograms of enantiopure RDA products (+)-**8**, (-)-**8**, (+)-**9** and (-)-**9** *S11*

1. Copies of ^{1}H- and ^{13}C NMR spectra of 4-16

***tert*-Butyl ((1*S*,2*S*,3*R*,4*R*)-3-(prop-2-yn-1-ylcarbamoyl)bicyclo[2.2.1]hept-5-en-2-yl)carbamate [(–)-4]**

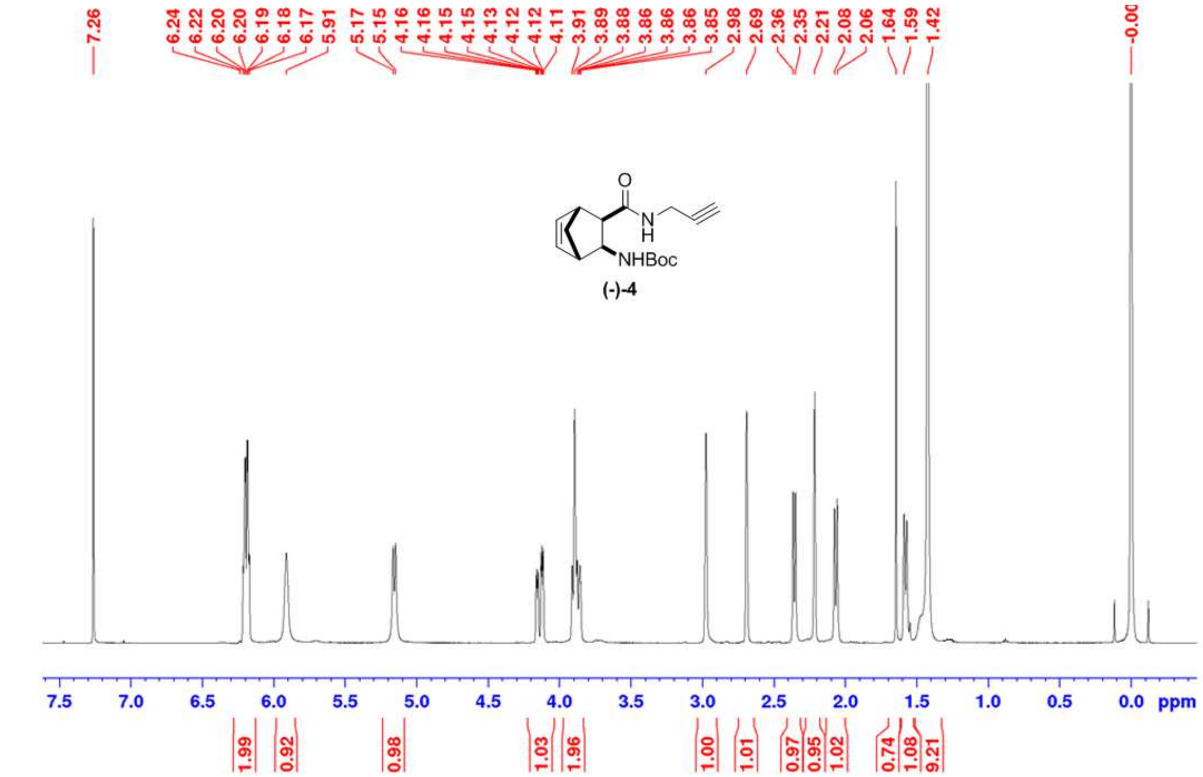

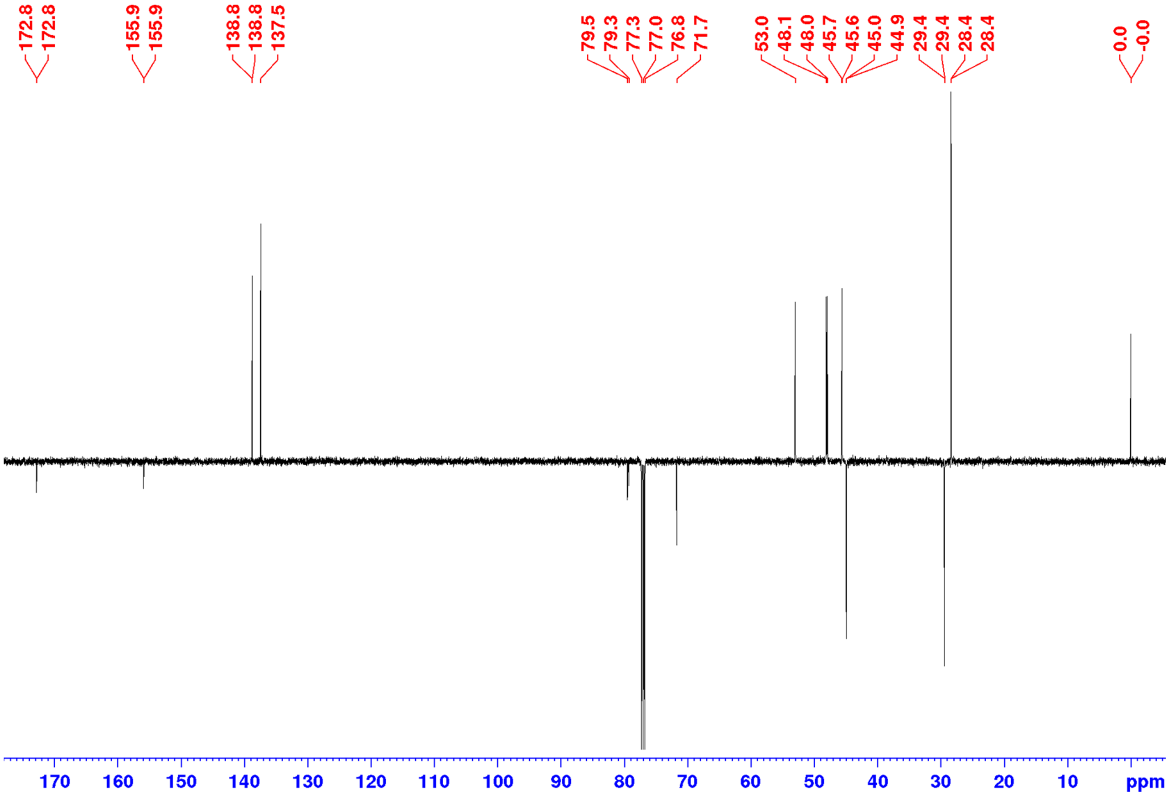

(1*S*,4*R*,4a*R*,6a*S*,12a*S*)-6-(Prop-2-yn-1-yl)-1,4,4a,6,6a,12a-hexahydro-1,4-methanoisoindolo-[2,1-a]quinazoline-5,11-dione [(–)-6]

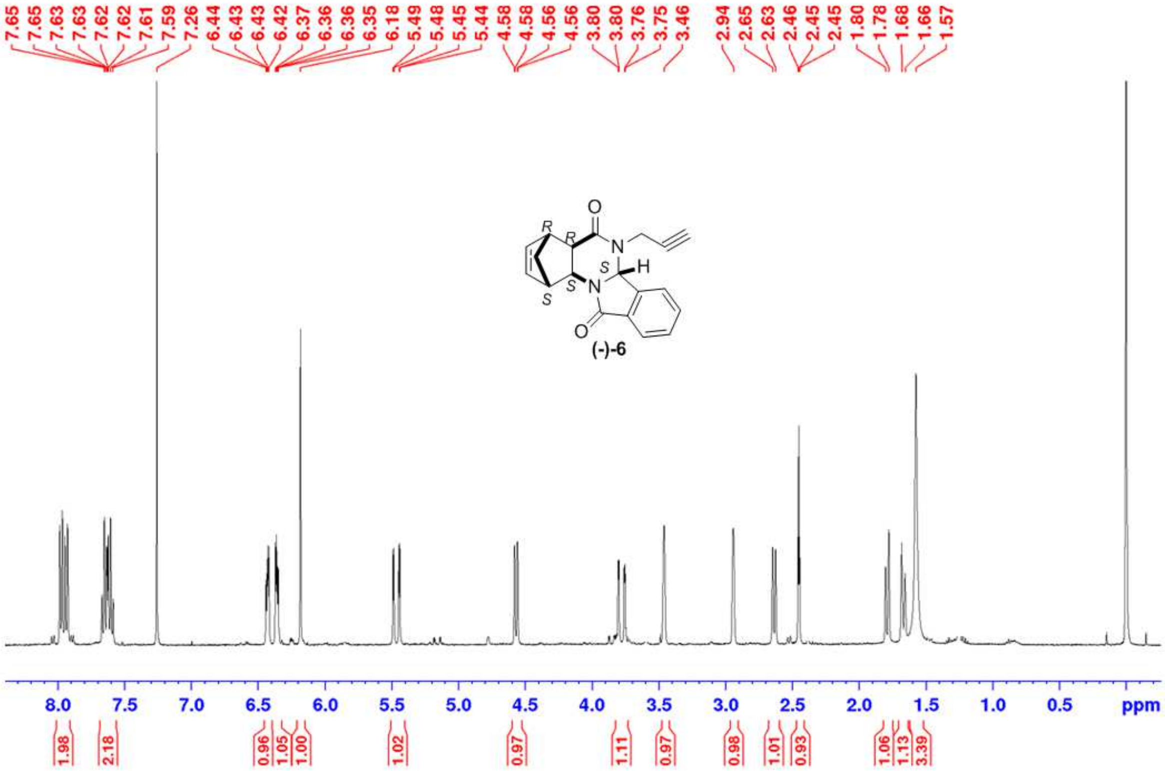

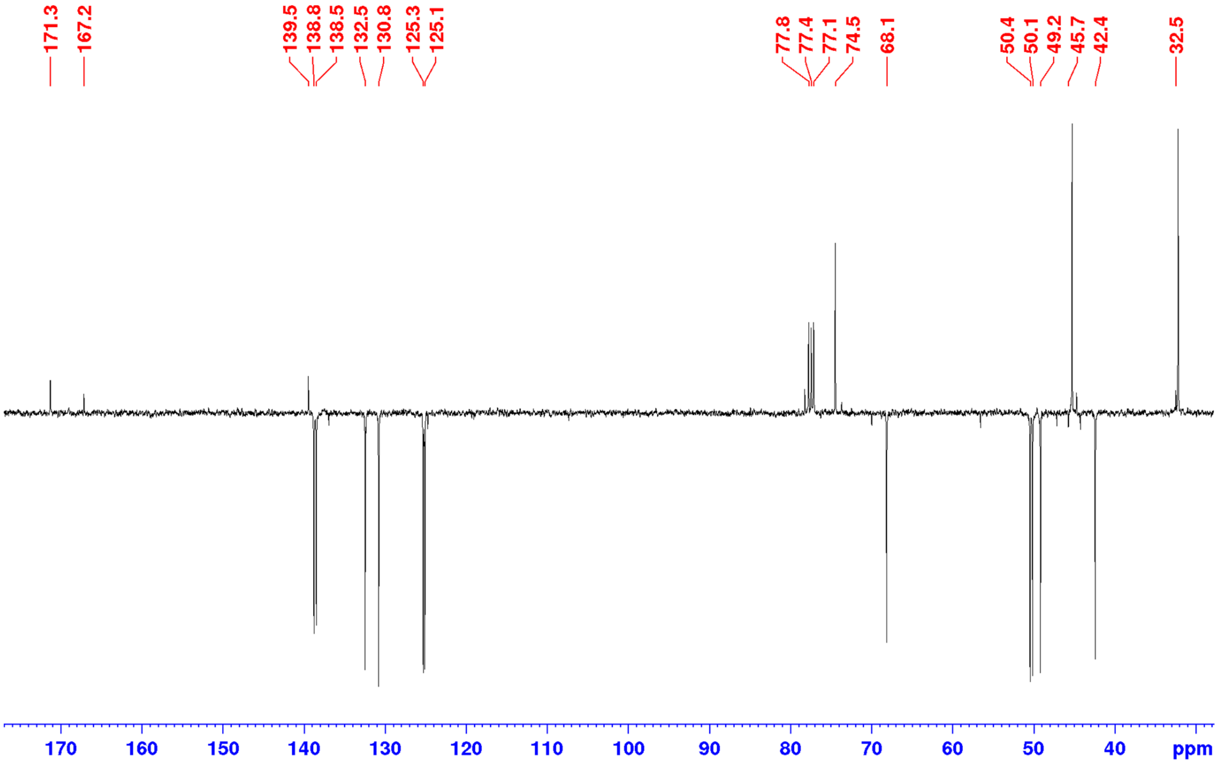

(1*S*,4*R*,4a*R*,6a*S*,12a*S*)-6-((1-(2-Methylbenzyl)-1H-1,2,3-triazol-4-yl)-methyl)-1,4,4a,6,6a,12a-hexahydro-1,4-methanoisoindolo[2,1-a]quinazoline-5,11-dione [(–)-7]

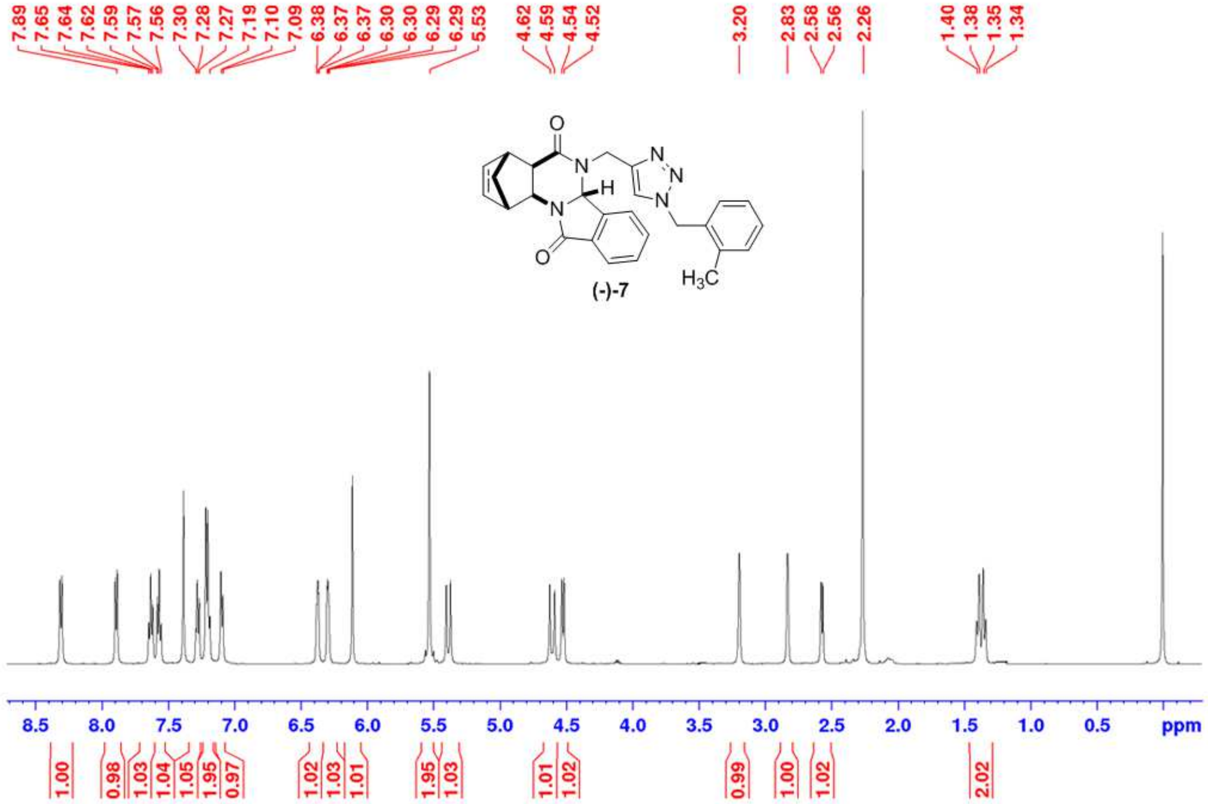

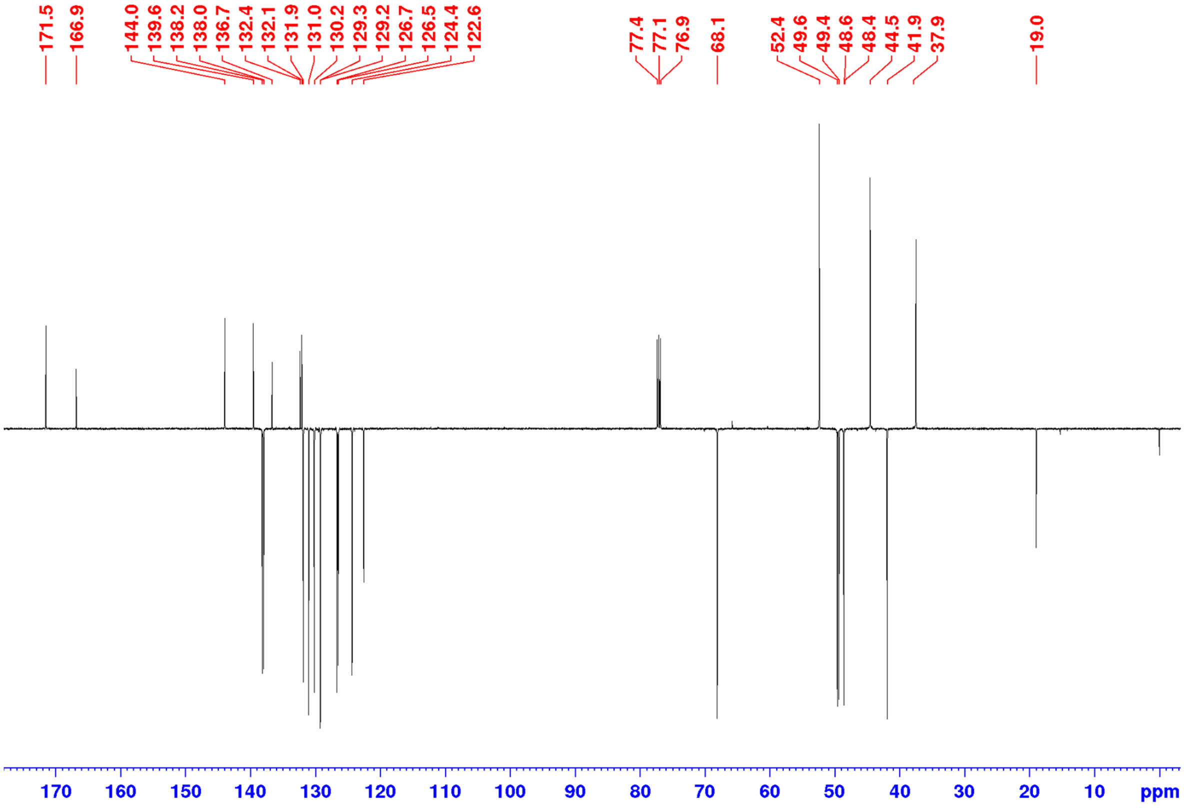

(S)-1-(prop-2-yn-1-yl)-1,10b-dihydropyrimido[2,1-a]isoindole-2,6-dione [(–)-8]

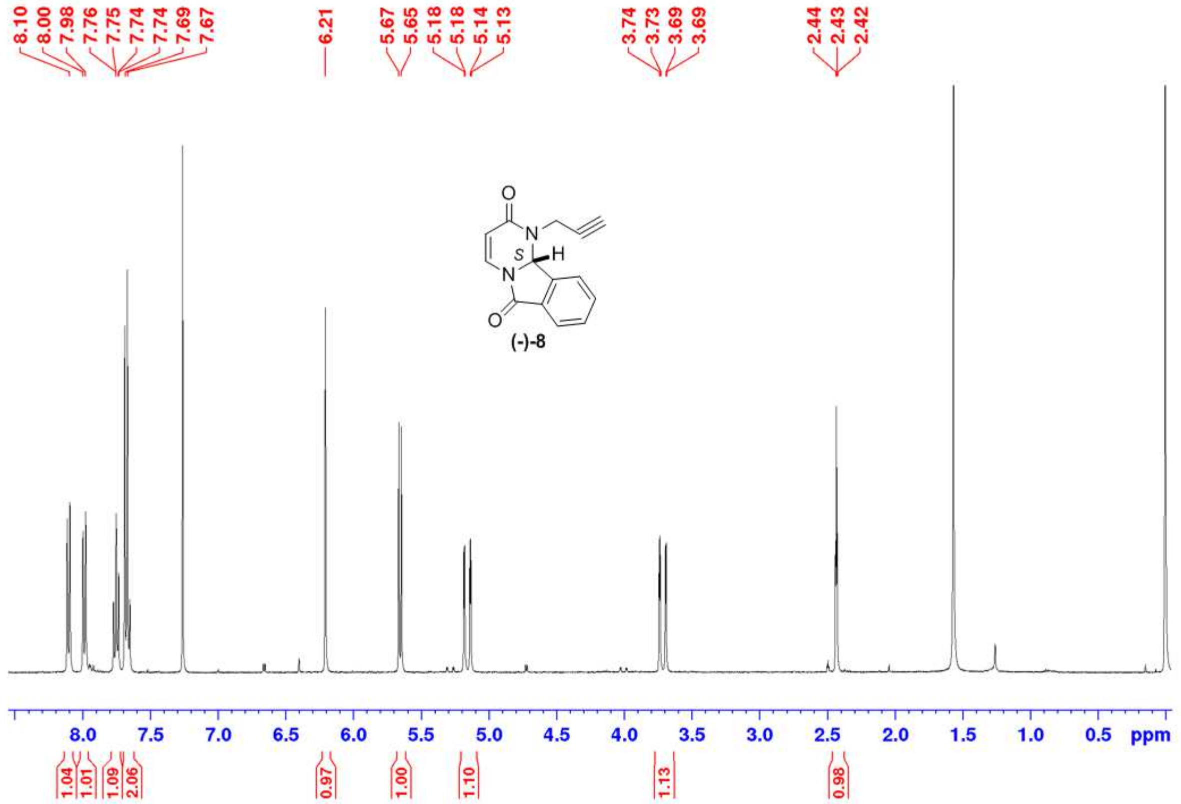

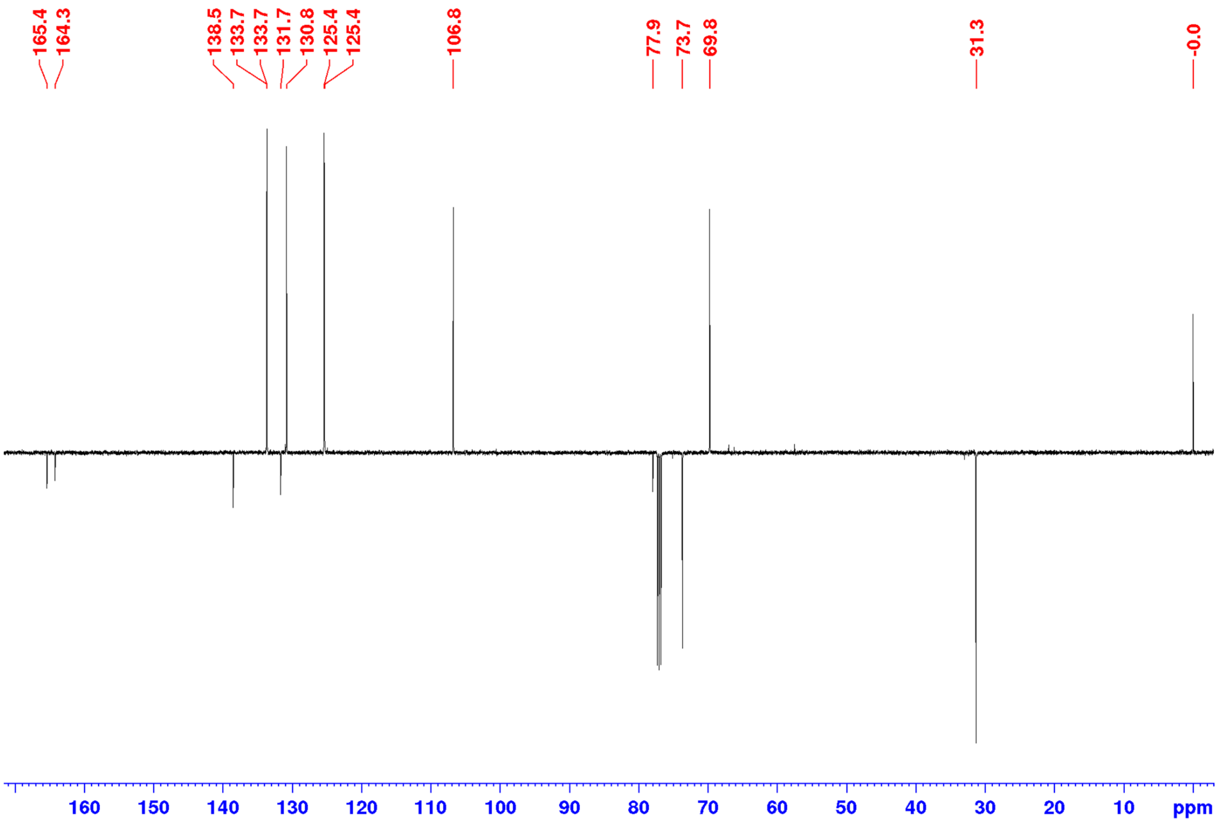

(S)-1-((1-(2-Methylbenzyl)-1H-1,2,3-triazol-4-yl)methyl)-1,10b-dihydropyrimido[2,1-a]isoindole-2,6-dione [(–)-9]

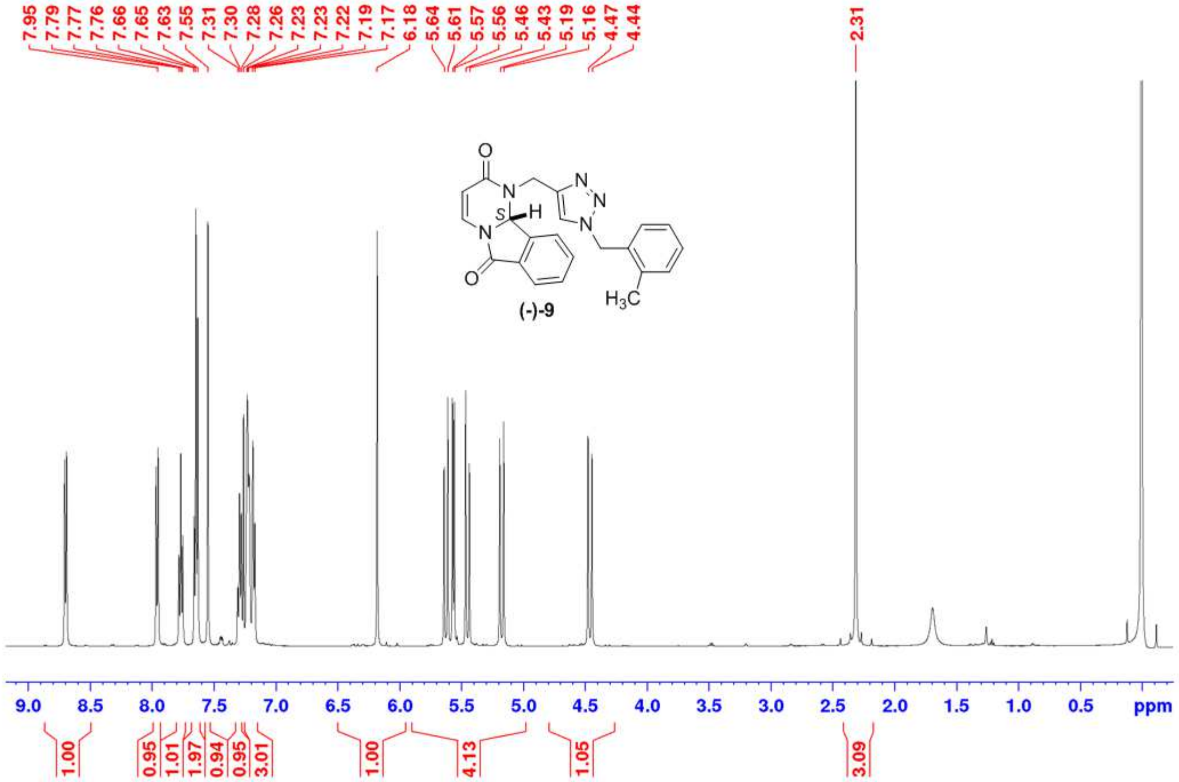

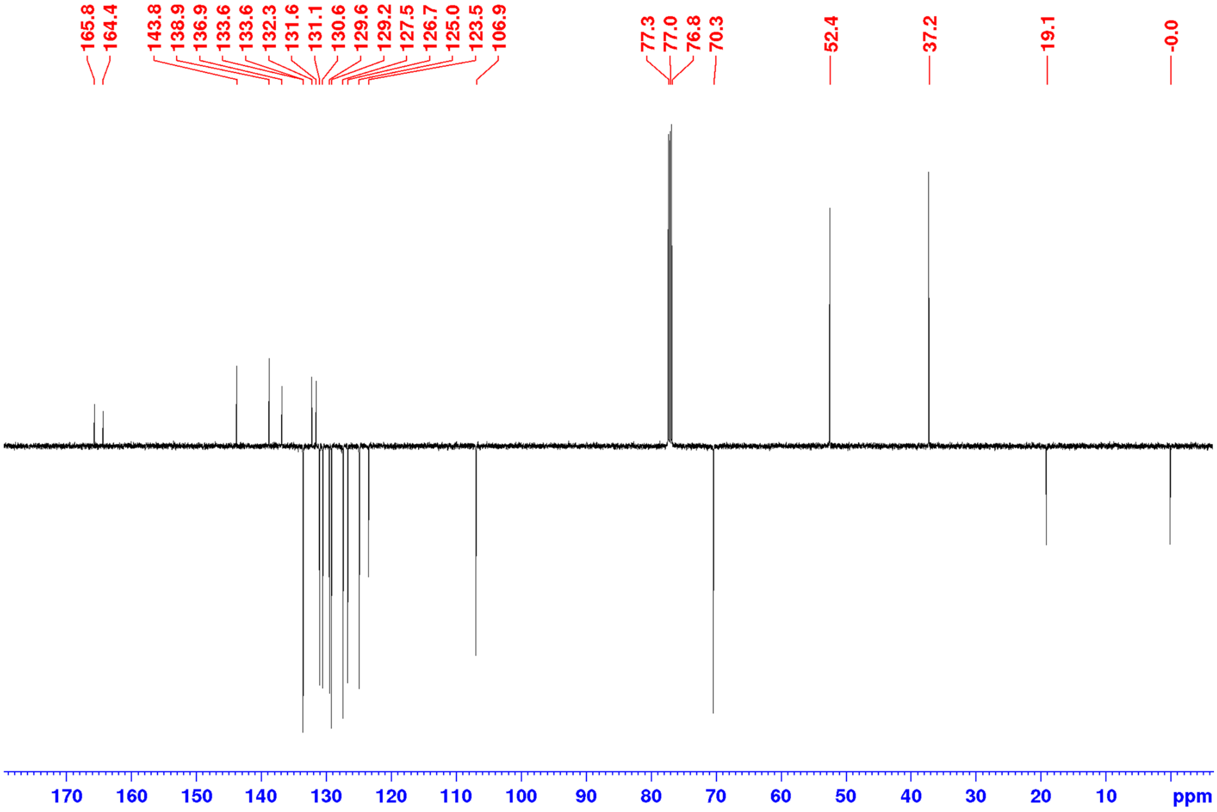

***tert*-Butyl ((1*R*,2*S*,3*R*,4*S*)-3-(prop-2-yn-1-ylcarbamoyl)bicyclo[2.2.1]hept-5-en-2-yl)carbamate [(–)-13]**

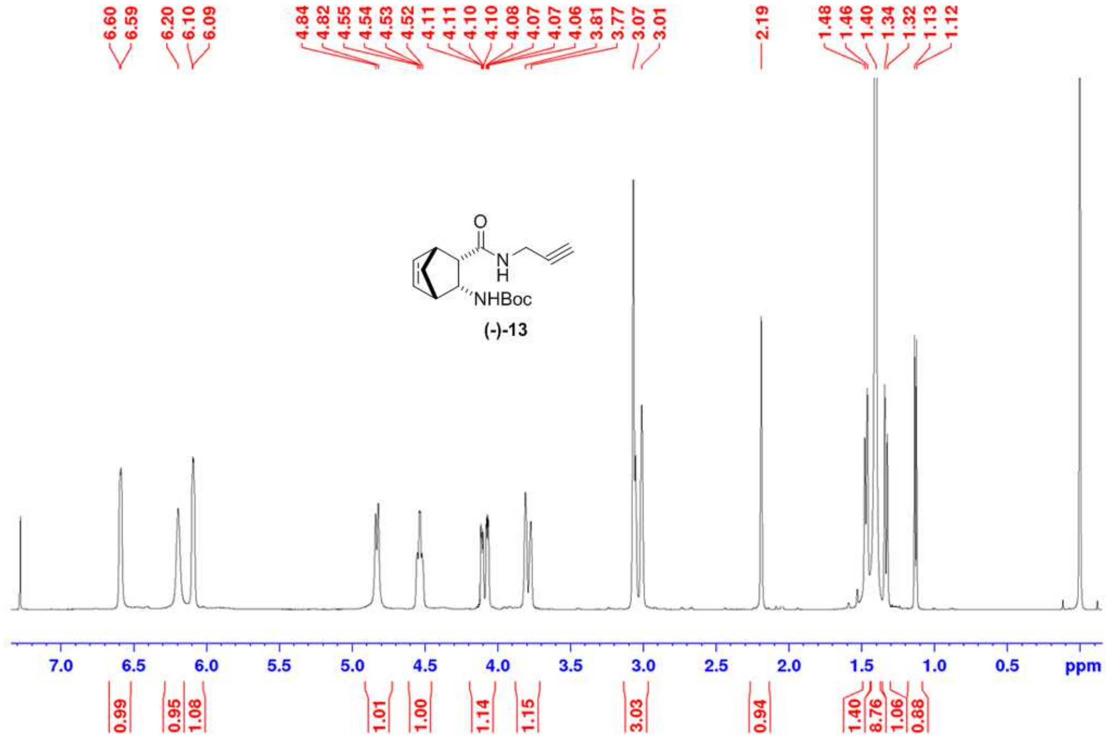

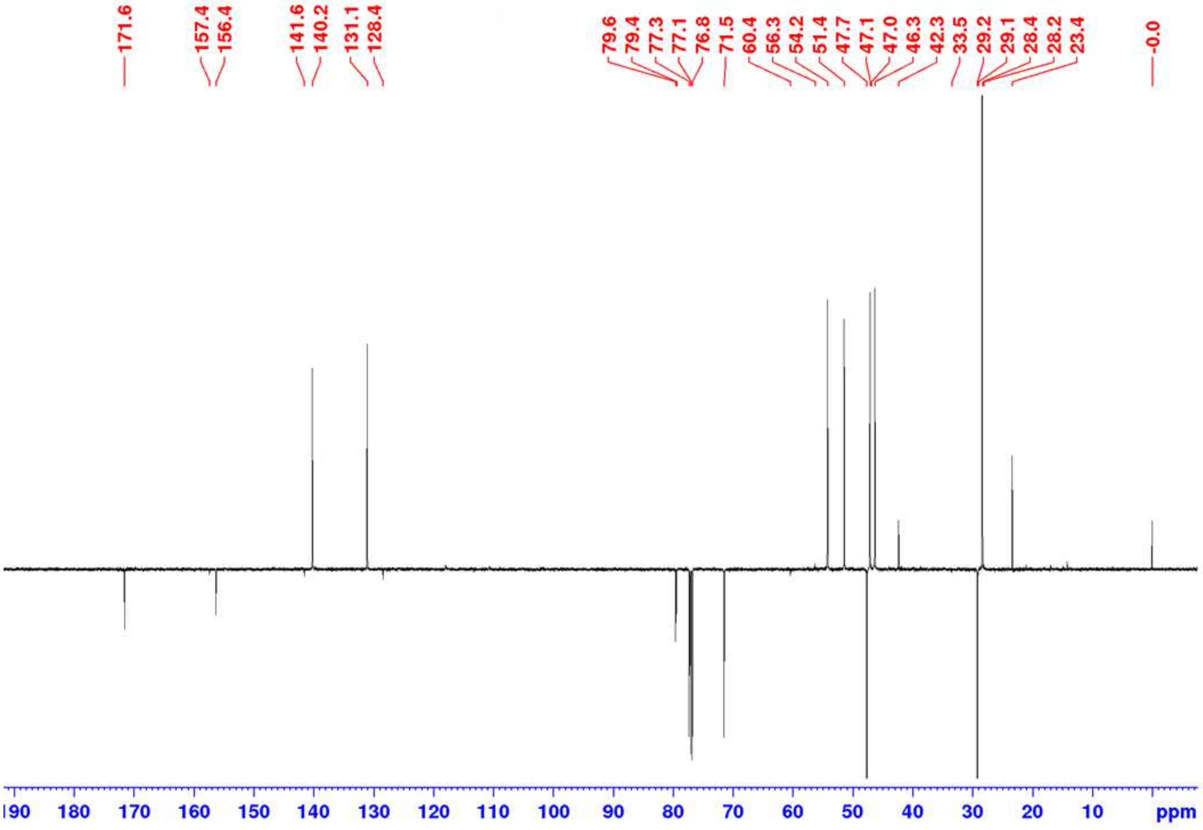

(1*S*,4*R*,4a*S*,6a*R*,12a*R*)-6-(Prop-2-yn-1-yl)-1,4,4a,6,6a,12a-hexahydro-1,4-methanoisoindolo-[2,1-a]quinazoline-5,11-dione [(–)-15]

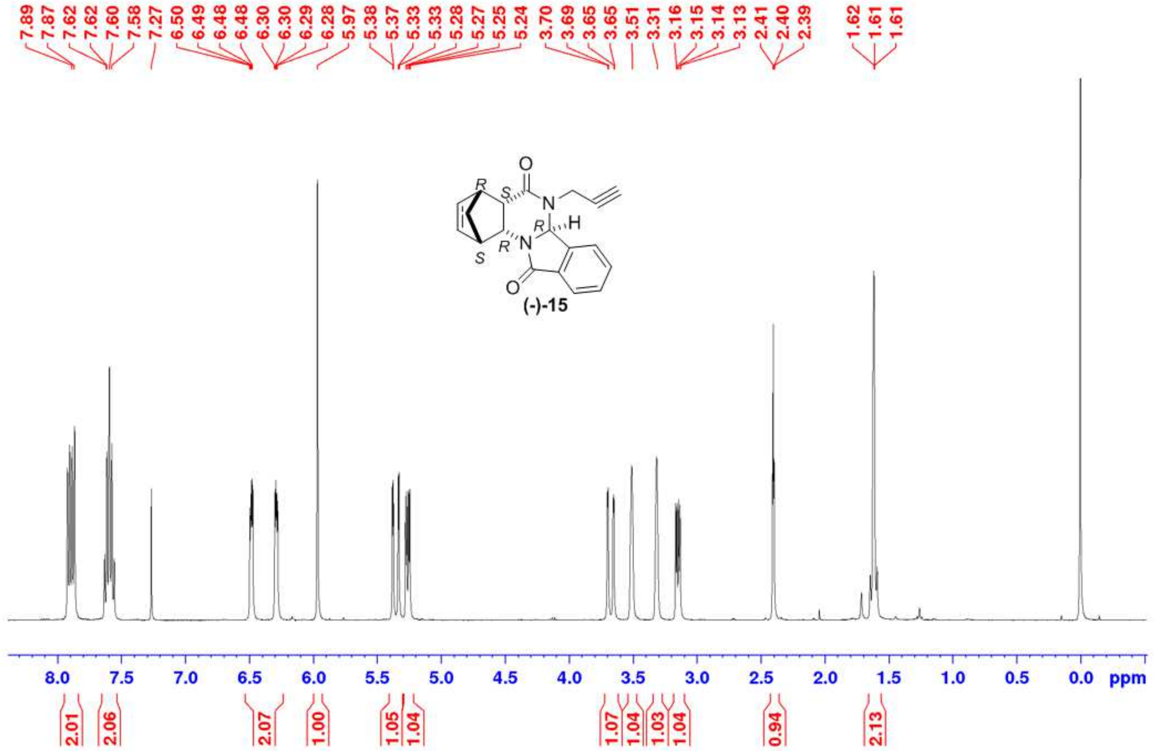

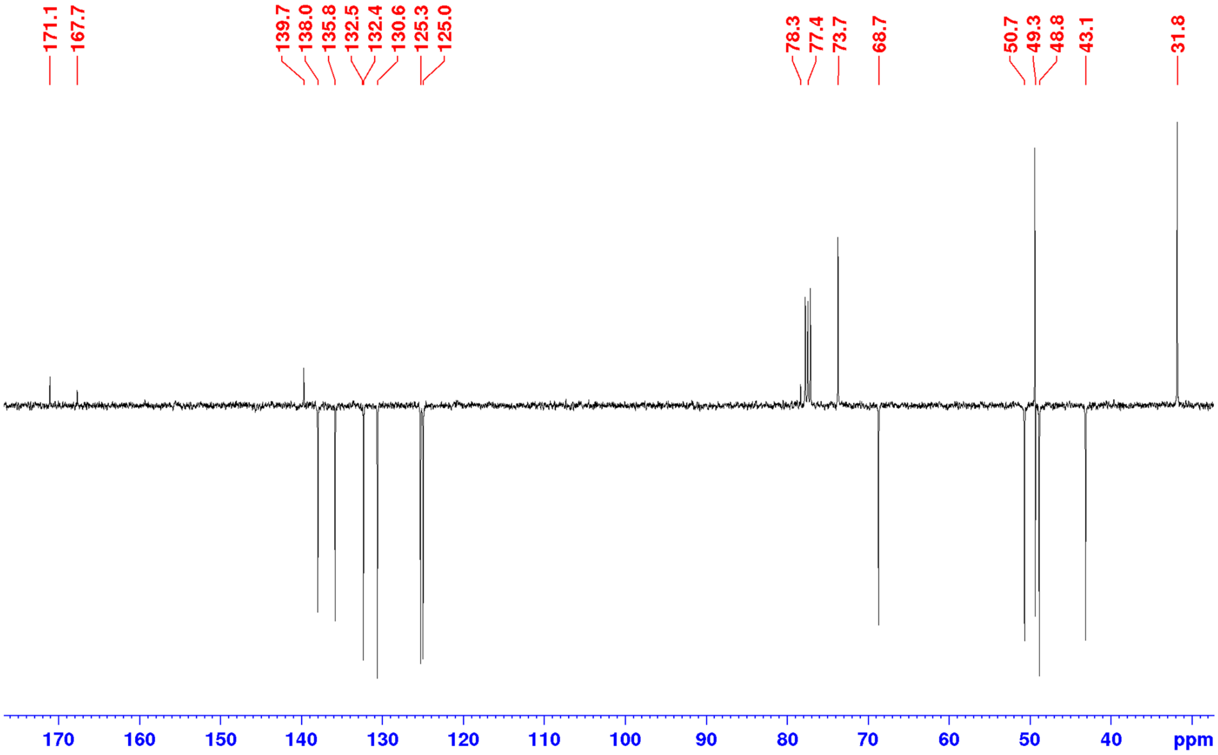

(1*S*,4*R*,4a*S*,6a*R*,12a*R*)-6-((1-(2-Methylbenzyl)-1H-1,2,3-triazol-4-yl)-methyl)-1,4,4a,6,6a,12a-hexahydro-1,4-methanoisoindolo[2,1-a]quinazoline-5,11-dione [(–)-16]

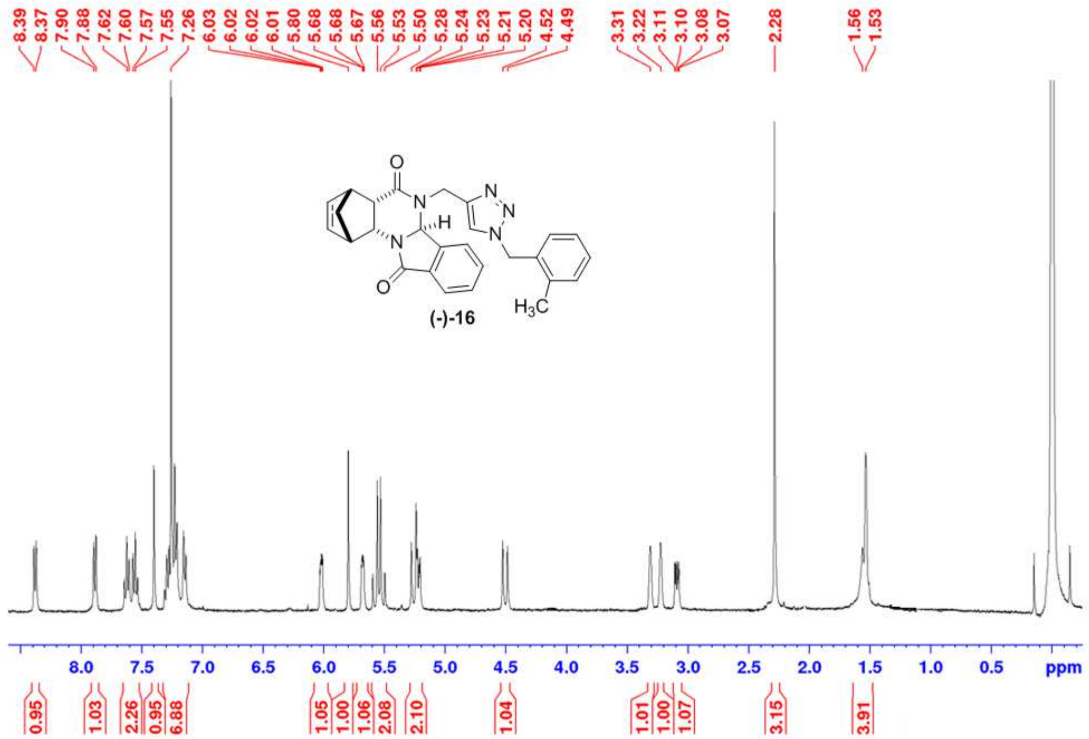

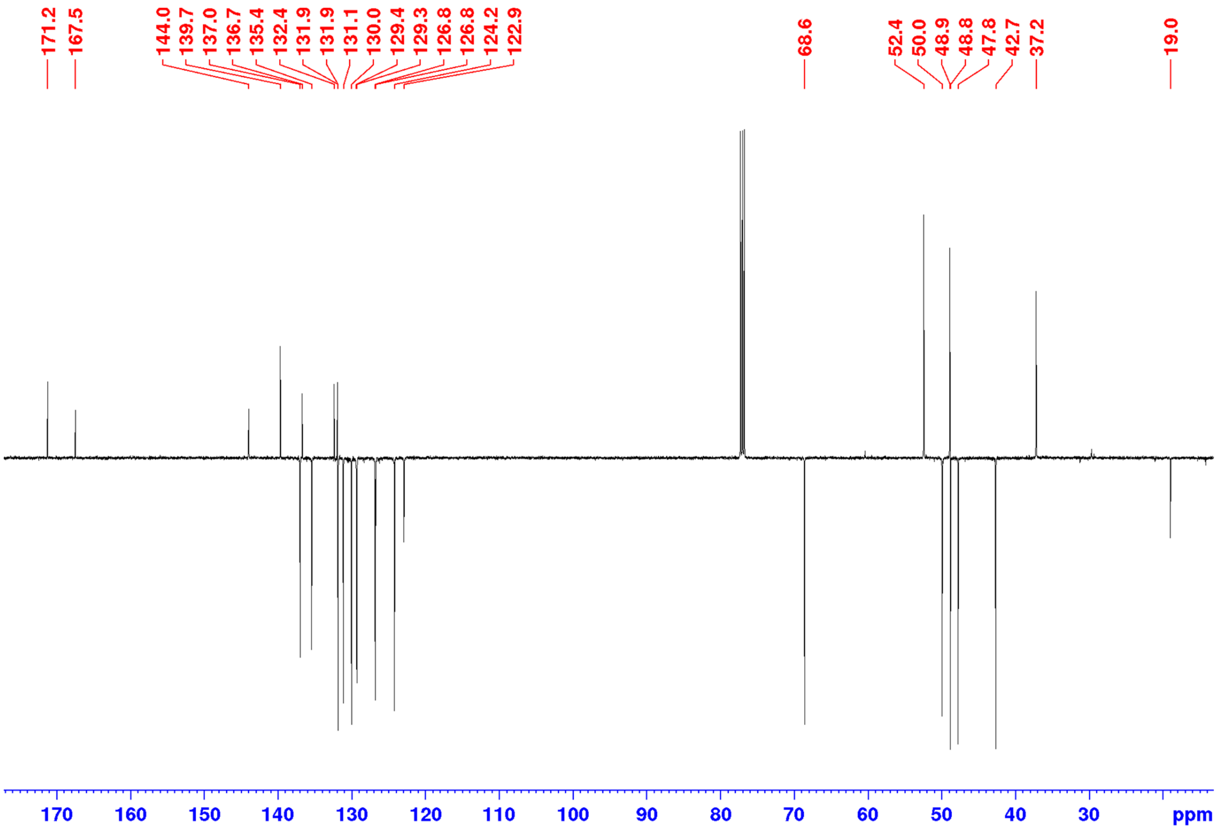

2. HPLC chromatograms of enantiopure domino products (+)-6, (-)-6, (+)-15 and (-)-15,

The *ee* values of the domino ring closure products (–)-**6** and (+)-**6**, were determined by HPLC using Chiracel-OD-H column, eluent: a mixture of *n*-hexane and IPA (70:30), flow rate: 0,15 mL min^{-1}, detection at 254 nm, retention times (–)-**6**: 61.83 min, (+)-**6**: 66.29 min.

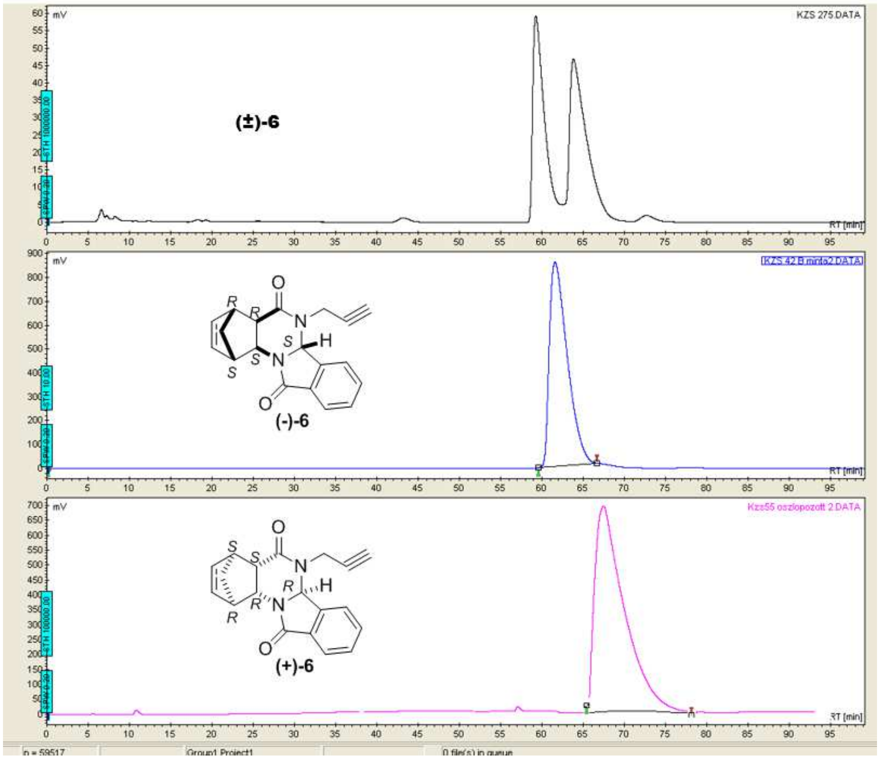

The *ee* values of the domino ring closure products (–)-**15** and (+)-**15**, were determined by HPLC using Phenomex-IA column, eluent: a mixture of *n*-hexane and IPA (60:40), flow rate: 1 mL min^{-1}, detection at 254 nm, retention times (–)-**15**: 22.04 min, (+)-**15**: 42.99 min.

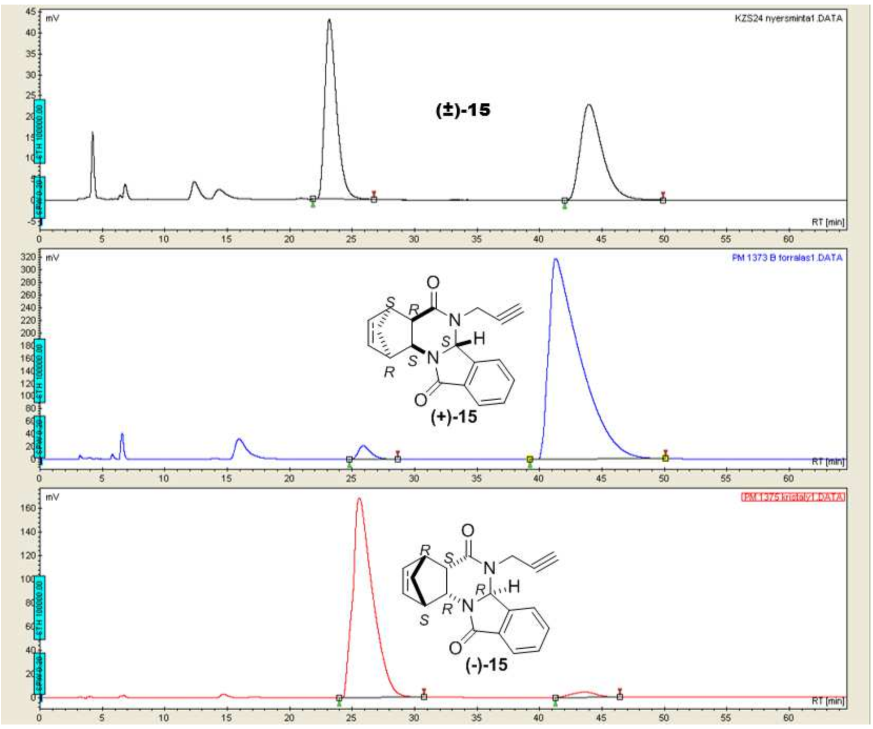

3. HPLC chromatograms of enantiopure RDA products (+)-8, (-)-8, (+)-9 and (-)-9

The *ee* values of the RDA products (–)-**8** and (+)-**8**, were determined by HPLC using Phenomex-IA column, eluent: a mixture of *n*-hexane and IPA (70:30 containing 0.1% DEA) flow rate: 0.5 mL min^{-1}, detection at 254 nm, retention times (–)-**8**: 80.88 min, (+)-**8**: 76.58 min.

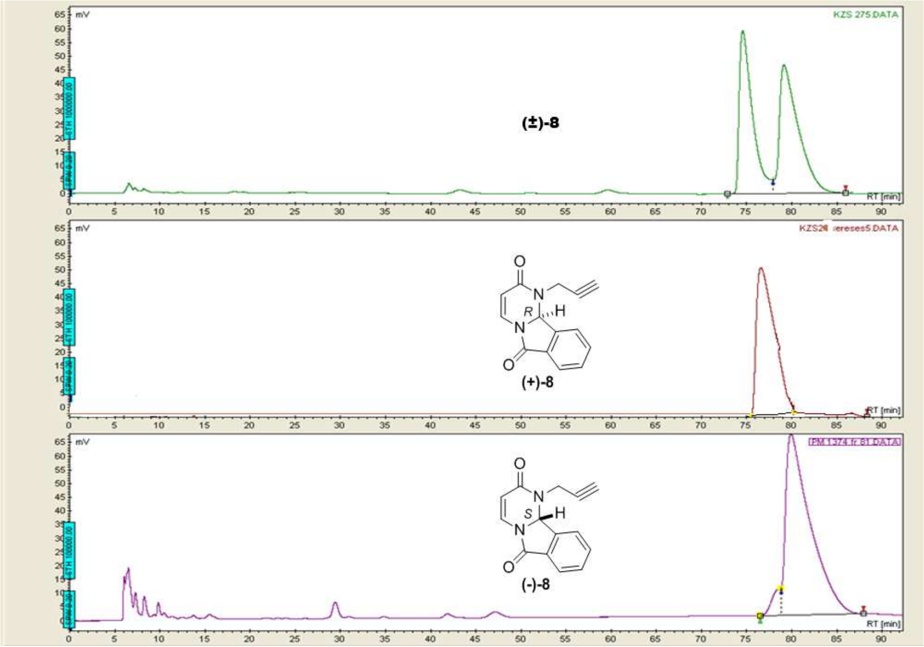

The *ee* values of the RDA products (–)-**9** and (+)-**9**, were determined by HPLC using ChiralPak-IA column, eluent: a mixture of *n*-hexane and IPA (60:40 containing 0.1% DEA) flow rate: 0.5 mL min^{-1}, detection at 254 nm, retention times (+)-**9**: 23.87 min, (–)-**9**: 26.76 min,.

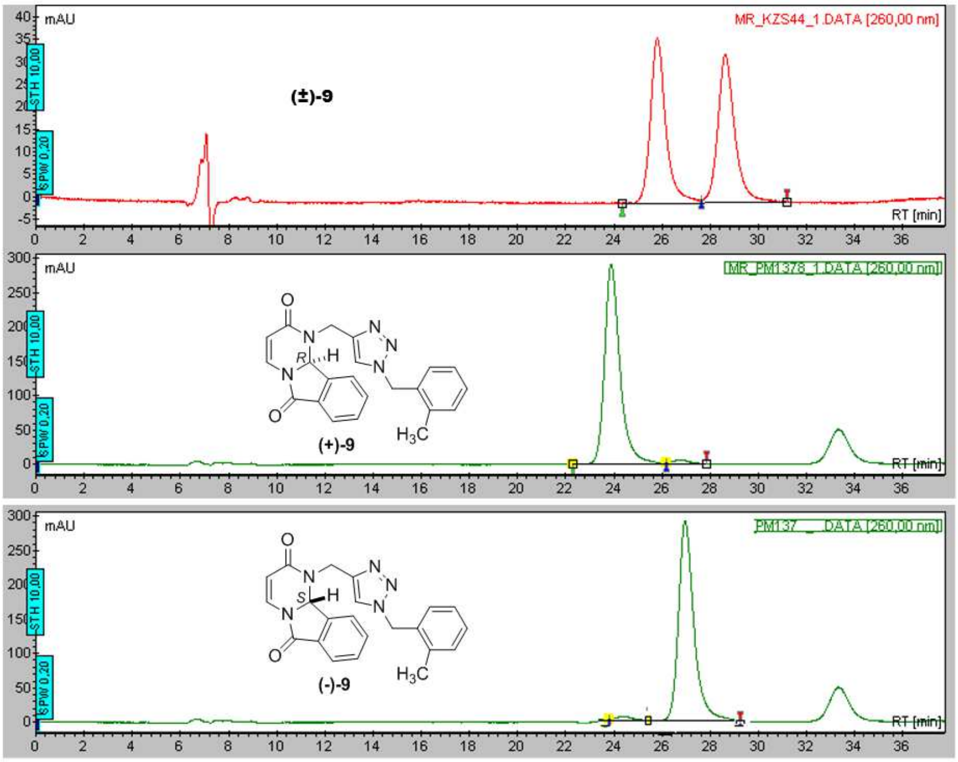

Supplement: Supplementary file 1 [file molecules-24-00772-s001.pdf]
